# Real-world insights of patient voices with age-related macular degeneration in the Republic of Korea and Taiwan: an AI-based Digital Listening study by Semantic-Natural Language Processing

**DOI:** 10.1186/s12911-025-02929-5

**Published:** 2025-03-18

**Authors:** Hyewon Jeon, Su-Yeon Yu, Olga Chertkova, Hyejung Yun, Yi Lin Ng, Yan Yoong Lim, Irina Efimenko, Djoubeir Mohamed Makhlouf

**Affiliations:** 1 Roche Product Development Safety Risk Management, Roche Products Pty Limited, Sydney, Australia; 2 Department of Pharmacy, College of Pharmacy, Kangwon National University, Chuncheon, Republic of Korea; 3 Roche Product Development Safety Risk Management, Roche Products Limited, Welwyn Garden City, UK; 4 Roche Product Development Safety Risk Management, Roche Korea Company Ltd, Seoul, Republic of Korea; 5 Roche Product Development Safety Risk Management, Roche (Malaysia) Sdn. Bhd., Subang Jaya, Malaysia; 6 Roche Product Development Safety Risk Management, Roche Hong Kong Limited, Kowloon Bay, Hong Kong SAR; 7 Semantic Hub SA, Lausanne, Switzerland; 8 Roche Product Development Safety Risk Management, F. Hoffmann-La Roche AG, Basel, Switzerland

**Keywords:** Macular degeneration, Natural language processing, Patient voice, Real-world data, Semantics/semantic analysis, Unmet needs, Tolerability, Digital, Social media

## Abstract

**Background:**

In this era of active online communication, patients increasingly share their healthcare experiences, concerns, and needs across digital platforms. Leveraging these vast repositories of real-world information, Digital Listening enables the systematic collection and analysis of patient voices through advanced technologies. Semantic-NLP artificial intelligence, with its ability to process and extract meaningful insights from large volumes of unstructured online data, represents a novel approach for understanding patient perspectives. This study aimed to demonstrate the utility of Semantic-NLP technology in presenting the needs and concerns of patients with age-related macular degeneration (AMD) in Korea and Taiwan.

**Methods:**

Data were collected and analysed over three months from January 2023 using an ontology-based information extraction system (Semantic Hub). The system identified patient “stories” and extracted themes from online posts from January 2013 to March 2023, focusing on Korea and Taiwan by filtering the geographic location of users, the language used, and the local online platforms. Extracted texts were structured into knowledge graphs and analysed descriptively.

**Results:**

The patient voice was identified in 133,857 messages (9,620 patients) from the Naver online platform in Korea and included internet chat forums focused on macular degeneration. The most important factors for AMD treatments were effectiveness (1,632/3,401 mentions; 48%), price and access to insurance (33%), tolerability (10%) and doctor and clinic recommendations (9%). Treatment burden associated with intravitreal injection of vascular endothelial growth factor inhibitors related to tolerability (254/942 mentions; 27%), financial burden (20%), hospital selection (18%) and emotional burden (14%).

In Taiwan, 444 messages were identified from Facebook, YouTube and Instagram. The success of treatment was judged by improvements in visual acuity (20/121 mentions; 16.5%), effect on oedema (10.7%), less distortion (9.1%) and inhibition of angiogenesis (5.8%). Tolerability concerns were rarely mentioned (26/440 mentions; 5.9%).

**Conclusions:**

Digital Listening using Semantic-NLP can provide real-world insights from large amounts of internet data quickly and with low human labour cost. This allows healthcare companies to respond to the unmet needs of patients for effective and safe treatment and improved patient quality of life throughout the product lifecycle.

**Supplementary Information:**

The online version contains supplementary material available at 10.1186/s12911-025-02929-5.

## Background

Age-related macular degeneration (AMD) affects almost 200 million people worldwide, is a leading cause of vision loss, and is expected to increase in prevalence with an aging population [1]. The global prevalence of AMD has been estimated to be 8.69% (95% credible interval 4.26% – 17.40%) [1]. In the Republic of Korea (hereinafter referred to as “Korea”), the prevalence of AMD is 13.9% (95% confidence interval 13.2% to 14.7%) [2], with AMD detected in about one-third of individuals aged 70 years or older. In Taiwan, the prevalence of AMD in individuals aged 70 years and over has been estimated to be 17.4% [3].

Treatment options for AMD include intravitreal injections of vascular endothelial growth factor (VEGF) inhibitors, thermal laser photocoagulation, and photodynamic therapy [4]. However, these treatments have been commonly associated with challenges such as cost, frequent clinic visits, and injection tolerability concerns [4].

Understanding the perspective of patients and caregivers, including their values, needs, and concerns, is crucial in achieving optimal outcomes for patients with AMD. Indeed, focusing on the patient voice can support the discovery of “pain points” with current treatments, the detection of unmet medical needs, and a deeper understanding of disease burden and its effects on quality of life [5]. However, currently, the voices of patients and their caregivers often remain unheard and underrepresented in treatment algorithms.

Traditionally, understanding the patient perspective involved labour-intensive, manual qualitative methods such as market research, interviews, surveys, and focus groups. However, even where quantitative analyses are conducted, these studies are limited by small sample size, selection, recall and reporting biases, lack of generalisability and/or representativeness, delayed data collection, and high cost [5].

Today, the World Wide Web offers an alternative source of patient perspectives, with abundant information provided in real-time on patient open chat forums and other open online platforms. Social Media Listening (SML) – monitoring and analysing discussions on social media platforms – is one of the methods of gathering rich information and has been used to complement existing, traditional methods for understanding the patient voice [5]. As the range of online platforms (YouTube, search engines, online forums, reviews, and communities) using interactive communication is now broader than conventional social media platforms, a broader methodology, which we have termed “Digital Listening” may be a more inclusive term.

While conventional SML is used with keyword search and manual human review of individual search results, Semantic-based Natural Language Processing (Semantic-NLP) is a form of artificial intelligence that analyses texts based on meaning. It can be a method to better generate insights without manual review, by understanding the searcher’s questions and the contextual meaning of terms to generate relevant results [6–11]. The insight extracted from patient stories shared online can be considered a new kind of real-world data, which leverages first-hand testimony that is not driven by a researcher. It is inclusive and non-discriminative, including voices of potentially underrepresented groups and covers aspects like barriers to treatment patients face or quality of life issues that are not available in other real-world data sources such as electronic health records. In addition, it can address the needs of caregivers.

Digital Listening can be used by pharmaceutical companies, with the encouragement of regulatory agencies, to better understand the patient perspective in the clinical development process [5] as well as post-marketing circumstances. This may ultimately enhance treatment outcomes through a timely response to patient needs that may otherwise have gone unrecognised. Given the patient voice can be influenced by factors such as the healthcare system and culture, country-specific patient insights may be required to allow tailoring of treatment, management, and collaboration with specific populations.

The objective of our research was to explore AI-based Semantic-NLP Digital Listening to gain comprehensive insights into the pain points, needs, and voices of patients with AMD in Korea and Taiwan.

## Methods

### Study design

We used Semantic-NLP to analyse real-world data obtained from internet posts describing the patient experience of AMD in Korea and Taiwan. Korea and Taiwan were selected due to their similar healthcare environments [12] including universal healthcare coverage with National Health Insurance, and similar access to pharmaceuticals, income levels and high internet usage.

Data collection occurred between 23 January 2023 and 27 January 2023 using the Semantic Hub platform (https://semantic-hub.com/), and semantic processing and data interpretation were carried out. Additional data collection following refinement of the model occurred from 20 March 2023 to 30 March 2023. The platform interrogated data that had been posted on the internet from 1 January 2013 to 19 March 2023. The analysis was finalised on 06 April 2023, with the study duration from data collection to data analysis being less than 3 months.

**Fig. 1:**
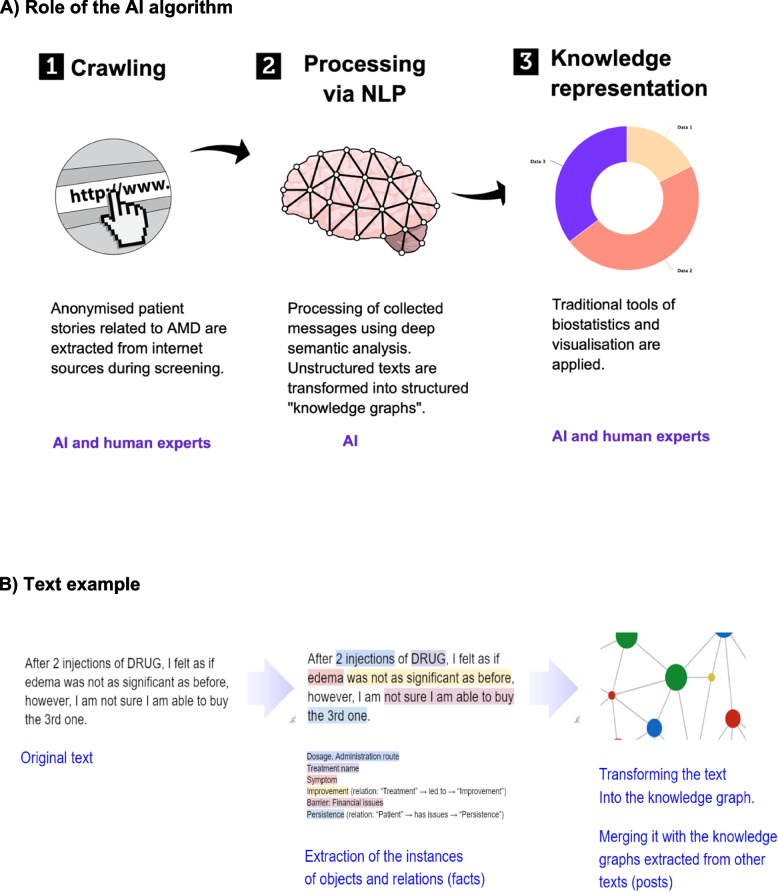
Process of identifying, extracting and presenting data. Note: Within the knowledge graph, the nodes are objects and the lines are relations between them (facts)

Semantic Hub technology transforms unstructured free text into a structured knowledge base, presented in the form of a knowledge graph. This technology belongs to ontology-based information extraction (OBIE) systems [13]. An overview of the process the algorithm uses to identify, extract and analyse relevant information is provided in Fig. 1.

Written natural language texts were analysed by their meaning and the context of the extracted “objects” and “facts”. The configuration of the ontology (what should be extracted) is defined on the level of the classes of objects (e.g. “a treatment name”, “a condition”, “a diagnosis” and “a symptom” as subclasses of “a condition”) and facts (e.g. a condition “is a side effect of” a treatment). By extracting objects and facts, analysis of natural language texts occurs using semantics, and not just keywords.

These Semantic-NLP modules are trained based on the corpora of texts whose qualitative and quantitative characteristics correspond to the features of texts analysed during the research project. The modules for each specific natural language (e.g. Korean, Traditional Chinese) are trained based on the corpora of texts in the corresponding languages. Training is carried out constantly as part of the development of the Semantic Hub platform, as well as at the launch of each specific research project. The training corpora includes more than 100 million texts and contains examples of the classes of objects and facts. Training procedures include fine-tuning machine learning modules, such as large language models, as well as the development of rule-based (pattern-based) modules for the extraction of objects and relations.

### Data source and collection

#### Internet crawling

Internet searching was conducted by the AI algorithm with the assistance of NLP engineers to tune the AI to relevant material. Generally, the crawling involved only open sources. However, the presence of closed data sources was also of interest as data extraction from such sources may be permitted with the approval of the relevant platform manager. Human experts were also involved to define and select the platforms to be screened for processing the specific sources. The AI system searched global platforms, including Facebook, X/Twitter, YouTube, and Instagram as well as local platforms, for both Korea and Taiwan. The data sources / websites were not strictly predetermined. Searches were conducted using two approaches: 1) the analysis of platforms actively used in countries of interest (as an example, Facebook for Taiwan and Naver for Korea), 2) additional searches were performed in the open Web environment.

To obtain data specific to Korea and Taiwan, the following three points were considered: 1) the geographic location of users, 2) the language used in the posts and 3) the internet portal environment. Geographic location, indicated by the names of cities and hospitals, was obtained from the content of messages and by checking the online profiles of users. Nuances in a local language, such as dialects and the use of traditional Chinese characters in Taiwan, were considered. Also, local online platforms, search engines, and local social media platforms were searched. Digital magazines and those of official bodies that have a purely informational nature were excluded from the search (i.e. the study was focused on the analysis of user-generated content to capture patients’ / caregivers’ messages, as well as on the detection of influencers).

The AI screened the identified sources for patient “stories” (the voice of the patient) related to conditions, symptoms, or treatments of interest. The stories identified by the AI algorithm were anonymized and collected in a database for further analysis. Since the research was oriented towards the detection of trends related to treatment value, success criteria for treatment, and safety and efficacy of treatment, the stories were categorised and generalised, and specific signals were considered as part of a category.

#### Selection of data for data extraction

The AI platform used deep semantic analysis to identify and extract themes of interest.

Included posts mentioned any of the following:

- Diagnosis of AMD;
- Mention of drugs that may be used to treat AMD such as ranibizumab (Lucentis), aflibercept (Eylea), brolucizumab (Beovu), bevacizumab (Avastin), faricimab (Vabysmo); anti-VEGF therapy. (A full list of drugs including biosimilars is in the Supplementary Table);
- Other diseases treated with the drugs of interest (diabetic macular oedema, proliferative diabetic retinopathy, vascular occlusions and ischaemia, retinal vein occlusion (RVO), myopic choroidal neovascularisation (MCNV), retinopathy of prematurity (ROP), neovascular glaucoma (NVG), central serous retinopathy (CSR), corneal neovascularisation);
- The abbreviations MD, CNV and other markers: patient with MD, age-related macular disease, lesion / dystrophy / macular degeneration, presence of reticular drusen, subretinal drusenoid deposits (SDDs), reticular pseudo-drusen (RPD), age-related maculopathy, macular lesion, dry / wet / neovascular / exudative dystrophy / retinal degeneration, retinal neovascularisation, formation or regression of choroidal neovascularisation (CNV), mentioning of a deposition of material in and on Bruch’s membrane.

In Taiwan, both English and Traditional Chinese drug names were used to detect the messages of interest. In Korea, searching was in Korean and English. Non-meaningful messages were excluded such as those expressing support for other users without mentioning any facts. Posts that mentioned central vision loss (stroke in visual cortex), eye injury, eye melanoma, or retinal haemorrhages were also excluded.

The identification of material for inclusion and exclusion was an iterative process. The initial set of concepts and terms was defined by human experts. Following this, the platforms on which patients / caregivers post their stories (messages) were selected using AI algorithms under the supervision of human experts, and messages of patients and caregivers containing the defined concepts were detected by the algorithm. Based on the analysis of such messages, additional inclusion and exclusion criteria were formulated (additional terms were included, other terms were excluded or modified). This sequence of steps was repeated several times until no new concepts or terms to be used as inclusion and exclusion criteria emerged in the newly collected messages. For example, it was found that patients rarely use the term ‘age-related macular degeneration’ specifically in both countries due to the lengthy disease term in local languages, so the search was widened to include ‘macular degeneration’.

The material identified as representing the patient's voice may have been written by a patient or their caregiver but will be referred to in this article as the voice of the patient, with caregivers considered to represent the opinions and concerns of the patients they care for. The patient's voice was distinguished from the voice of the healthcare professional based on the semantics used in the post.

### Data analysis

The extracted unstructured texts were transformed into structured ‘knowledge graphs’, which allowed the application of data visualisation and biostatistics. Human analysts, i.e. data scientists and country experts, were involved with the analysis and interpretation of the knowledge graphs.

Descriptive analyses were performed. Categorical variables are presented as proportion of messages, rather than proportion of patients, to further protect patient anonymity. However, age and gender were counted only once if included in multiple messages from a single internet account. Anonymous posts were considered to have been posted by different people.

Where it was completely missing, age and gender were not imputed. If age was referred to indirectly as a period of life, it was imputed. For example, 40 years old was used for an unspecified age in the 40 s, 42 years old for ‘early 40 s’, 45 years old for ‘mid-40 s’ and 47 years old for ‘late 40 s’.

We specially analysed the following aspects:

- Description of included patients, selection criteria of physician or treatment, patient expectations and concerns about the treatments, treatment burden, and treatment persistence.

## Results

### Korea

#### Description of included patients

**Fig. 2:**
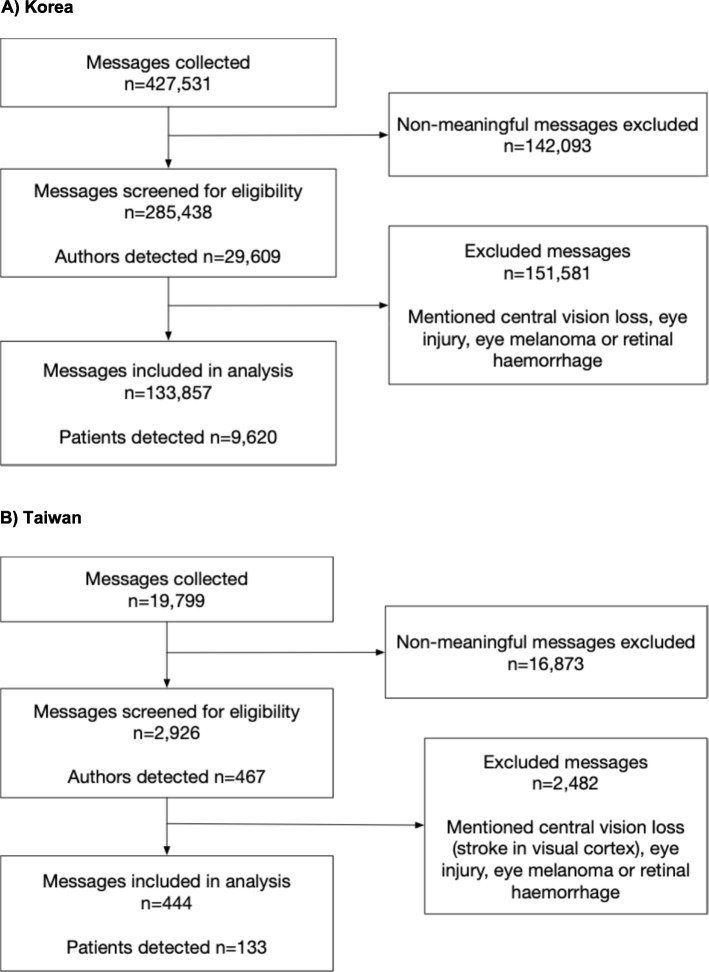
Selection of messages in the study. Note: A much higher proportion of messages collected in Taiwan (85%) than in Korea (33%) were excluded for having non-meaningful content. This may be explained by the availability of relevant online support groups for patients and carers in Korea, while in Taiwan there were general groups for the discussion of eye health and with a significant number of advertisements

The patient's voice was identified in 133,857 messages that came from the Naver online platform (Fig. 2) which included several internet chat forums focused on macular degeneration. The AI system also searched global platforms for relevant patient or caregiver messages in Korea. However, these platforms did not yield substantial patient-related content for this region. Instead, most of the community discussions were concentrated on local platforms like Naver. Some YouTube channels and other resources such as University hospitals or doctor’s blogs appeared in the AMD search. However, they were excluded from the analysis because they were not the patient’s or caregiver’s messages. No data was extracted from closed data sources.

The 133,857 messages represented the voices of 9,620 patients (Table 1). 8,506 used “MD” (macular degeneration) or related terms, and 564 patients mentioned having AMD. Among patients who stated their gender (*n* = 3,557), the numbers of males and females were similar (47.5% and 52.5%, respectively). Age information was provided by 1,661 patients, with those under 50 years more likely to disclose their age than those aged over 50 years (67.0% vs 33.0%). Geographic locations were mentioned 7,329 times, with Seoul being the most cited city (20.1%), followed by Busan (6.7%), Daegu (6.0%), Suncheon (5.9%) and Seongnam (5.8%).

**Table 1:** Characteristics of messages and patients in Korea and Taiwan

	**Korea**	**Taiwan**
Total Messages (n)	133,857	444
Main platform source	NAVER^a^	Facebook, YouTube and Instagram
Total Patients (n)	9,620	133
Gender (%)		
n	3,557	95
Female	52.5	51.6
Male	47.5	48.4
Age (%)		
n	1,661	68
< 40	44.6	30.9
40 s	22.4	27.9
50 s	13.5	17.6
60 s	10.9	16.2
70 s	5.3	4.4
> 80 s	3.3	2.9
Location (%)		
n	7,329	67
Capital city	20.1	43.3

^a^Local platform in Korea

#### Selection of treating physician, treatment centre, and treatment

**Fig. 3:**
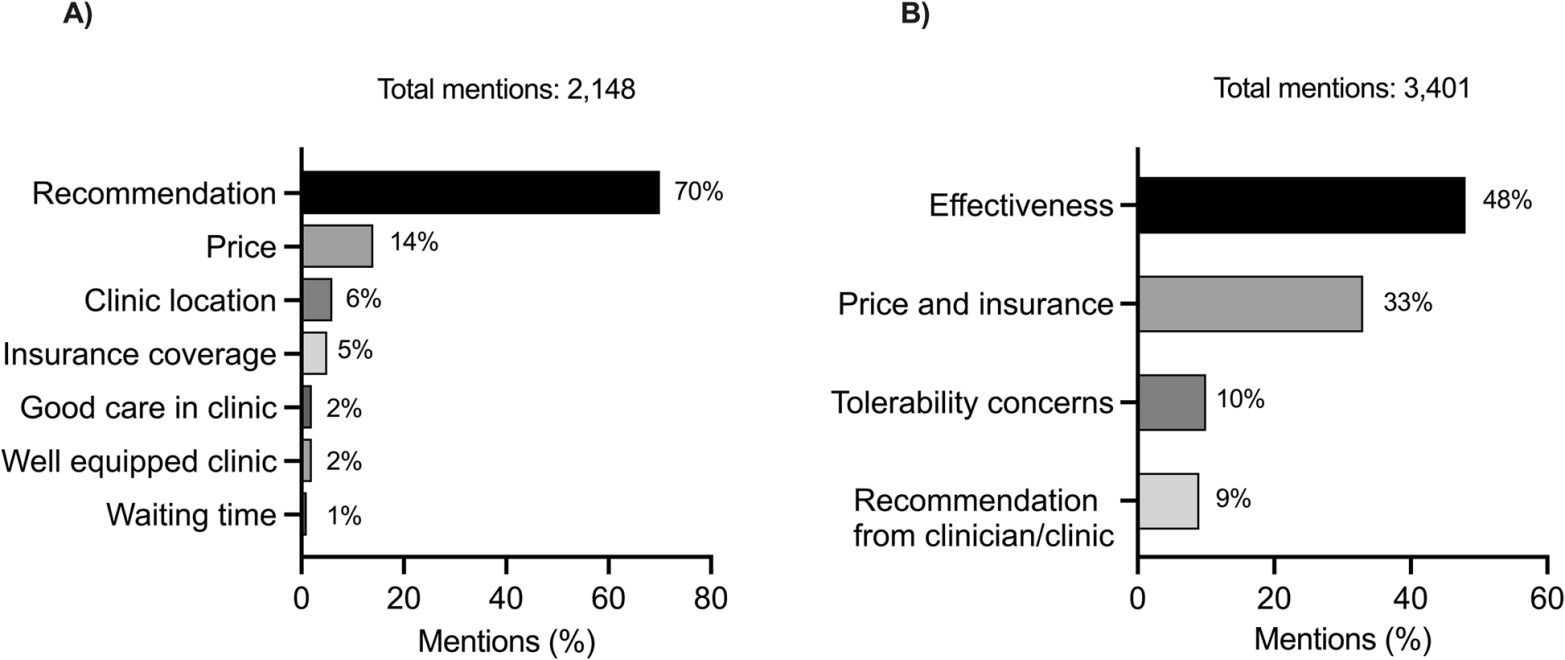
Criteria for selection of **A** clinician and treatment centre and **B** treatment in Korea

Of 2,148 mentions relating to the choice of physicians and hospitals, 70% indicated following a recommendation from the internet community, including other patients, caregivers, or healthcare professionals. 14% mentioned the price of injections, surgery, and diagnostic procedures as an important factor in their decision (Fig. 3A).

Out of 3,401 mentions discussing treatment selection criteria, 48% emphasised treatment effectiveness. Effectiveness was evaluated based on the speed and extent of improvement post-injection, duration of effects, and absence of noticeable benefits. Other commonly mentioned selection criteria were price and access to insurance (33%), tolerability issues (10%), and recommendations from their doctors and clinics (9%) (Fig. 3B).

#### Patient expectations and concerns about treatment

**Fig. 4:**
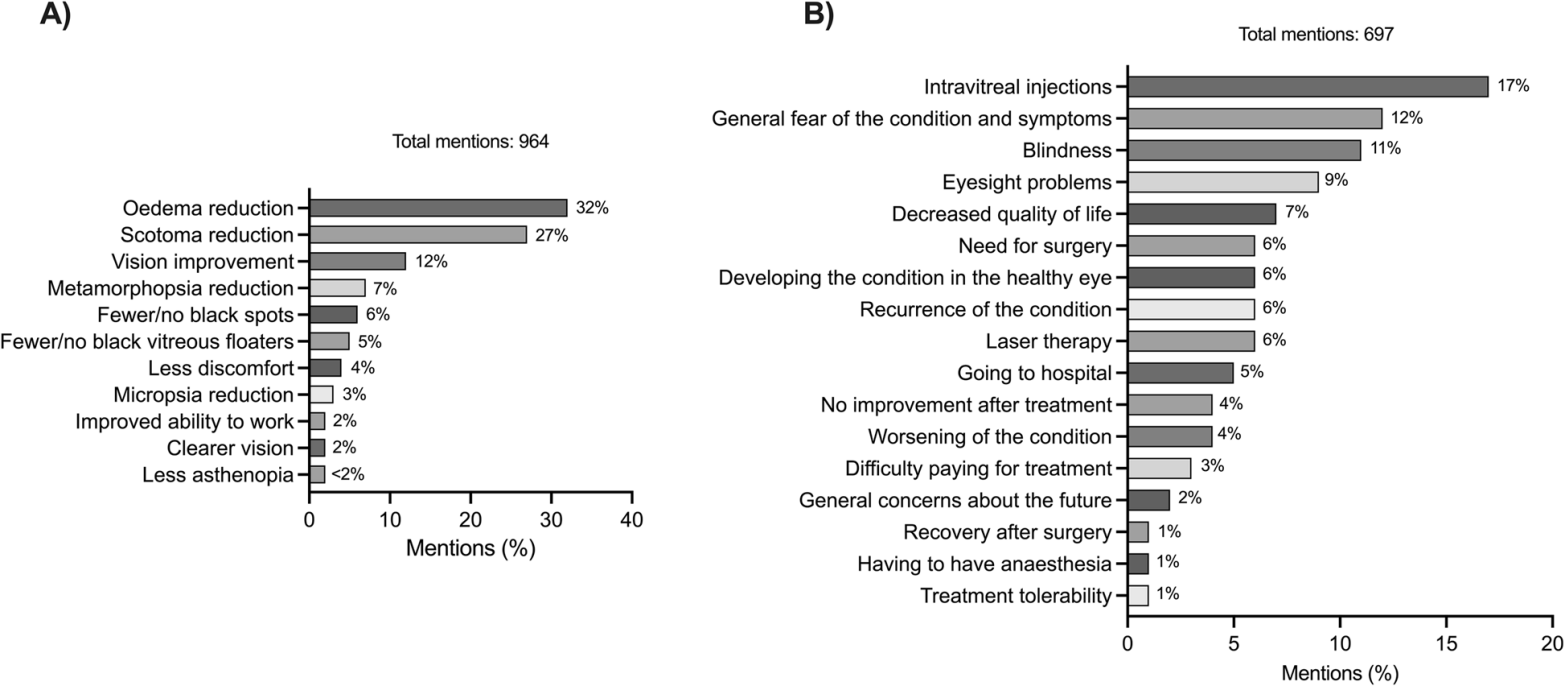
**A** Criteria patients use to determine the success of their treatment and **B** patients’ concerns about treatment outcomes expressed by patients in Korea

Discussion on treatment outcomes comprised 964 mentions, with the drainage of oedema (“water is gone”) being the most positively noted effect post-intravitreal injection. Patients expected improvements in symptoms like scotoma, visual loss, black spots, or vitreous floaters post-treatment (Fig. 4A).

On the other hand, 697 mentions expressed fears or concerns regarding treatment, including receiving intravitreal injections, progress of disease and its symptoms, blindness, eyesight problems, decreased quality of life, and surgery (Fig. 4B).

#### Treatment burden

**Fig. 5:**
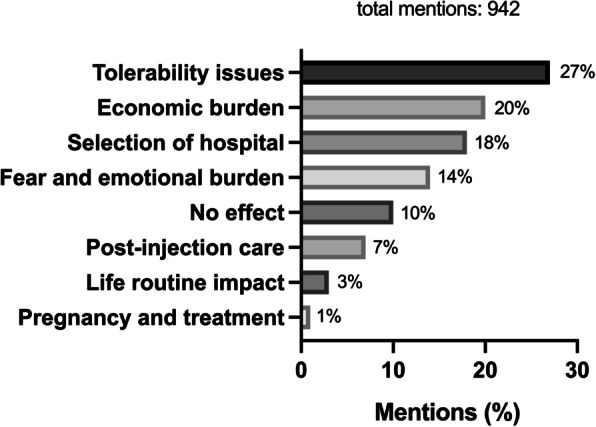
Treatment burden in Korea

There were 942 mentions of the burden of treatment, primarily relating to tolerability (27%), financial burden (20%), hospital selection (18%), and emotional burdens (14%) (Fig. 5).

There were 100 mentions of specific tolerability concerns. The majority related to visual loss, with other tolerability concerns including partial sight, unspecified eye symptoms, scotoma, vitreous floaters, and inflammation. Rarely mentioned tolerability concerns were eyeball soreness, phosphene, pain, elevated intraocular pressure, and discomfort.

There were 180 mentions of strategies followed if tolerability issues occurred. 77% continued therapy despite challenges, 13.3% switched therapy, and 9% discontinued the treatment. Fear of blindness (40.6%) and worsening conditions (37.2%) were significant reasons for continuing therapy.

Among patients aged less than 50 years, 21% complained about the high price of treatment and 79% were open to discussing the cost of treatment. Patients would consider paying out of pocket for anti-VEGF therapy as they had a strong fear of becoming blind or of their condition worsening. The least expensive originator anti-VEGF drug treatment was mentioned in 69.3% of relevant comments by patients under 50 years and 47.3% of comments from patients aged 50 years or older.

#### Treatment persistence/adherence

Four thousand two hundred and fourteen (4214) mentions described either persistence or adherence to treatment. 80.6% described receiving 1 or 2 injections, 13.8% described 3 to 6 injections, and 2.1%, 1.7%, and 1.6% described receiving 7 to 9, 10 to 14, and 15 or more injections, respectively.

Among patients who described the first or second injections, 20.6% switched to another anti-VEGF therapy, suggesting switching to anti-VEGF therapy happened in the early stage of treatment.

Patients were likely to continue taking treatment even if they had a negative experience (ineffective or tolerability concerns) or else switch treatment to another anti-VEGF therapy.

The reasons for treatment discontinuation (286 mentions) were primarily due to ineffectiveness (36%). However, 17.5% indicated patients ceased treatment after noticing certain symptom improvements.

### Taiwan

#### Description of included patients

The patient voice was identified from 444 messages that came from 118 patients and 15 caregivers (total = 133, Table 1). The majority of the messages came from Facebook, YouTube and Instagram. There was only one community on Facebook dedicated specifically to macular degeneration. It was newly created, with low engagement at the time this project began and became a closed site requiring permission to extract data. No data was extracted from this site. Out of 48 patients who mentioned their conditions, 46 patients (95.8%) stated they have MD, and only 2 patients (4.2%) stated that they have AMD.

Gender information was available for 95 patients, with males accounting for 48.4% and females for 51.6%. Patient age was obtained for 68 patients. 41.1% of patients mentioned their age as 50 years and above. Geographic locations were mentioned a total of 67 times, with Taipei being the most frequently cited city (43.3%). Other commonly mentioned cities included Kaohsiung, Taichung, Taoyuan and Hsinchu.

#### Selection of treating physician, treatment centre and treatment

Overall, hospital names were mentioned 52 times in patient posts (52/440 mentions; 12%). There were also 28 mentions of specific doctor’s names (6%). However, the main criteria considered related to physician selection were mentions of reputation or prominence, professional skills and experience in treating AMD, of which there were few mentions. Selection criteria for treatment were not discussed.

#### Patient expectations and concerns about treatment

**Fig. 6:**
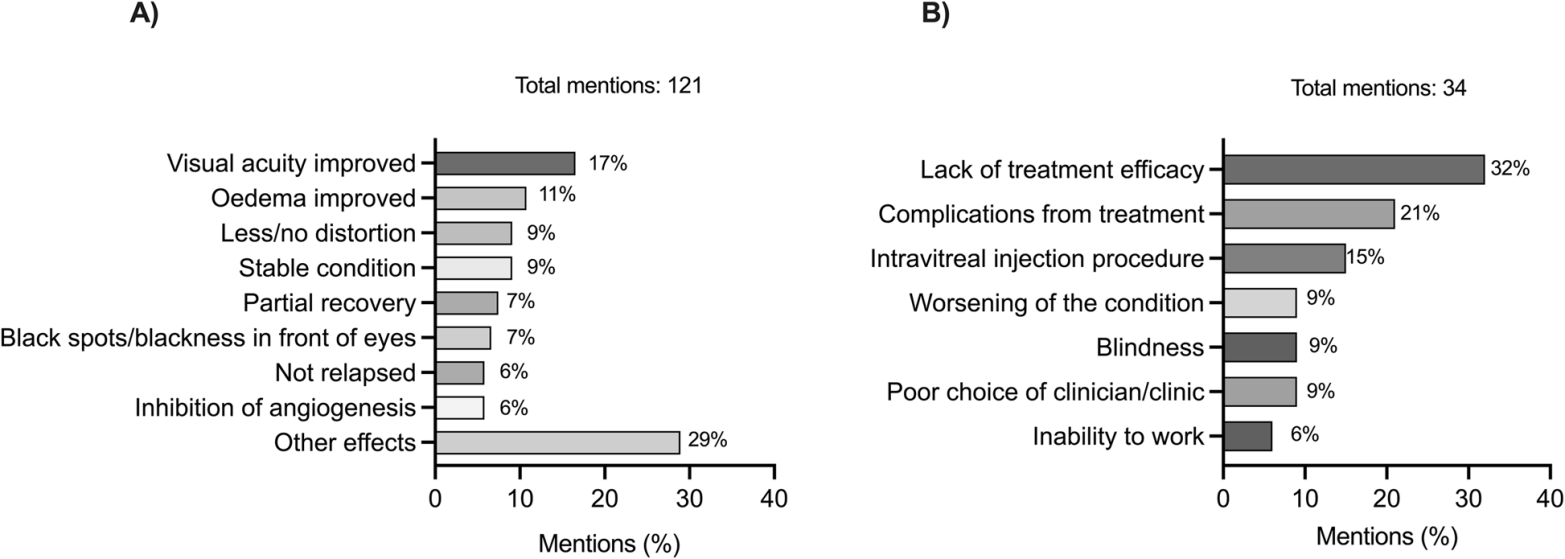
**A** Criteria patients use to determine the success of their treatment and **B** patients’ concerns about treatment outcomes expressed by patients in Taiwan

The success of treatment was judged by improvements in visual acuity (20/121 mentions; 16.5%) in Fig. 6A. Patients described this as gaining better vision, going from not being able to see to having clear vision and having clear sight over long distances. The other top treatment success criteria included reduction in oedema (10.7%), no or less distortion (9.1%), and staying stable or no regression (9.1%).

There were 34 mentions of fears and concerns. Patients worried most about lack of treatment effectiveness, followed by complications resulting from injections, finding a good doctor and clinic, worsening of the condition, and pain from injection (Fig. 6B).

#### Treatment burden

There were 32 mentions of disease and injection burden, with the majority of them relating to depression or psychological burden caused by the disease, followed by the length of the treatment process (spending a lot of time on treatment process/hospitals).

There were only few mentions of tolerability concerns related to anti-VEGF injections such as pain, and occasionally that they have small wounds, endophthalmitis or that they have watering eyes. Patients also discussed complications related to surgery, vitrectomy, laser, and photodynamic therapy.

Financial burden was the leading factor for non-persistence/non-adherence to treatment, in turn causing switching (e.g. to a less expensive treatment option or traditional Chinese medicines), treatment break or discontinuation. Discussions about the possibility and method of obtaining reimbursement, as well as the implications of discontinuing treatment were identified.

#### Treatment persistence/adherence

There were more than 70 mentions related to persistence and adherence. They were measured either in the number of injections, or number of months the patients were receiving treatments. In terms of the number of injections, 20.6% described receiving 1 to 3 injections, 7.3% described 4 to 5 injections and 11.8% described receiving more than 10 injections. In terms of treatment duration, 17.6% followed up on a regular basis, 10.3% followed up for 2 to 4 months, and 10.3% were unknown.

The first positive treatment experience was often achieved after 4 to 5 injections, and after 3 months of treatment.

### Comparison of patient preferences and treatment concerns between Korea and Taiwan

Although the data from Korea and Taiwan are not directly comparable, Table 2 highlights differences detected in patient treatment preferences and concerns between Korea and Taiwan.

**Table 2:** Comparison of patient preferences and treatment concerns: Korea vs. Taiwan

	Korea	Taiwan
Physician selection	Recommendation from others 70%, price 14%, clinic location 6%, insurance cover 5%, other 10%	Reputation / prominence; professional skills; experience in treatment of AMD (qualitative analysis due to a small data sample)
Treatment selection	Effectiveness 48%, price and insurance 33%, tolerability concern 10%, recommendations from doctors 9%	Not available
Treatment expectation	Oedema reduction 32%, scotoma reduction 7%, vision improvement 12%, metamorphopsia reduction 7%, other 22%	Visual improvement 17%, oedema reduction 11%, less distortion 9%, stable condition 9%, other 54%
Treatment concern	Intravitreal injections 17%, fear 12%, blindness 11%, eyesight 9%, other 51%	Ineffectiveness 32%, complication 21%, intravitreal injections 15%, worse condition 9%, other 23%
Treatment burden	Tolerability 27%, economic 20%, hospital selection 18%, emotional 14%, other 21%	Psychological 28%, treatment length 25%, injection burden 19%, economic 13%, other 15%

Note: The four most mentioned factors are listed, and other factors have been combined. In some cases, the ‘other’ categories represent a large proportion of the sample indicating a wide variety of responses

## Discussion

In our analysis of data from Korean and Taiwanese patients with age-related macular degeneration (AMD) and their caregivers, there were various challenges in making direct comparisons between the two countries. Firstly, there are discrepancies in data feasibility, such as differences in dataset size and sources. Korea (patients *n* = 9,620, messages *n* = 133,857) had a much larger dataset than Taiwan (patients *n* = 133, messages *n* = 444). Secondly, linguistic factors may play a role, such as using more or less precise terminology when describing a disease. Thirdly, cultural and social differences likely influenced the results. Korean patients and caregivers actively use dedicated online communities to share detailed and candid concerns. In contrast, Taiwan lacks such a local online community and may share information less publicly. Consequently, information from Korea was more diverse and detailed than that from Taiwan. There was little information on physician selection from Taiwan and no data on treatment selection.

Where data for Taiwan was available, we observed differences in patient preferences and treatment concerns between the two countries. Korean patients described success of treatment not just in terms of improvement in visual acuity but also as an improvement in the anatomy and physiology of the eye. This may indicate a higher level of patient education about AMD in Korea than in Taiwan. Korea also appears to have more active research interest in AMD, reflected in a high number of publications in AMD [14] and more comprehensive data available on the domestic distribution of AMD e.g. [15–18].

Our findings from quantitative digital listening were comparable with the findings from qualitative surveys in patients. A small survey of 17 European patients with AMD found that most were satisfied with their treatment despite fearing intravitreal injections [19]. Most patients were motivated to continue treatment; however, patients said that they may want to stop treatment if they felt it had no effect if the side effects of treatment outweighed the benefits, if their doctor told them to stop or if treatment costs were no longer reimbursed. They also mentioned that difficulties in travelling to and from appointments would reduce their ability to continue treatment. Drivers of and barriers to adherence were investigated in a multinational telephone survey of 94 patients, 79 caregivers, and 62 retina specialists conducted in North America and Europe [20]. The top barriers to patient treatment adherence were related to tolerability and fear of injection, followed by frequency of visits, inadequate education about treatment purpose, its risks and the procedures involved, travel logistics, poor doctor-patient relationship, and overall treatment effectiveness. While generally patients’ motivations and concerns were similar to those we detected through our Digital Listening, travel challenges were not mentioned in our research. This is possibly because patients have greater accessibility to medical institutions in Korea and Taiwan compared to the countries included in the previous studies.

A Chinese study of 21 patients attending face-to-face interviews from May 2018 to September 2018 investigated the experience of patients with AMD in the treatment decision-making process [21]. Patients weighed factors including blindness, the pain associated with intravitreal injections, family influence, financial burden, and assistance from professionals and peers. Constraints on patient decision-making included doctors who do not consider patient choice, inadequacy of options, and time to make decisions.

There are limitations in our study. Firstly, there exists a potential reporter bias as patients motivated to share their experiences online may disproportionately represent those with particularly positive or negative treatment viewpoints. Also, younger patients and those with higher visual acuity tend to participate more actively in the digital environment compared to older patients or those with lower visual acuity, which may introduce selection bias. Our data indeed includes a higher proportion of younger patients, with 67% (1113 patients) of Korean patients being under 50 years old and 59% (40 patients) in Taiwan, whereas AMD mainly occurs in older people. Therefore, our findings may not fully reflect the voices of the average AMD patient population. It is worth noting, however, that our study findings were largely consistent with other studies of patients with AMD and their caregivers [19, 20].

Secondly, unsolicited and secondary data are often incomplete, making it difficult to determine accurate patient demographics or details of data. Additionally, patients’ voices from outside the typical AMD demographic may have been captured because we selected and extracted data using broader terminology, including MD as well as AMD to account for patients who may not explicitly mention their exact diagnosis. In Korea and Taiwan, patients often use the shorter term "MD" instead of the full term "AMD," which led us to adopt this wider terminology for data collection. Nevertheless, much higher numbers of patients were identified in Korea (1,661 patients for age and 3,557 for gender) and included in this study than in previous qualitative AMD studies. In case the data detail is needed for a certain research purpose, further access to the messages for interest topics can be considered to complement the NLP analysis by ensuring data privacy and regulatory compliance.

Thirdly, the limited number of posts discovered from patients in Taiwan can raise concerns about the conclusiveness of our analysis for this country. Taiwan appears to have low engagement in digital activities, with digital communities often being closed forums (not open to the public). We found one active patient community describing macular disorders on Facebook with 77 followers; however, we chose not to include this data source as it was a closed group requiring consent to collect personal data from this site.

Our study has four main strengths. Firstly, this is the first study using Semantic-NLP to identify the perceptions of patients with AMD. We have showcased an efficient and robust method for generating insight from a very large data source using AI technology (i.e. large-volume data collection and analysis within 3 months for both countries). Further, as our AI platform analyses data using sources without human access to individual-level information and personal data, this approach enables healthcare companies to focus more efficiently on the primary purpose of the study, such as to generate collective insights of patients’ perceptions.

Secondly, despite the similar digital literacy level and age distribution of AMD/MD patients between Korea and Taiwan, our study highlights inter-country differences by applying the same Digital Listening methodologies. While both countries show high internet usage (98% in Korea in 2023 [22] and 91% in Taiwan in 2024 [23]), the volume and sources of data collected in this study were significantly different. In Korea, data were collected from local internet platforms providing support to patients with macular degeneration and their caregivers, with patients actively sharing information and opinions. In contrast, data from Taiwan was collected from non-local digital platforms that were not specifically aiming to support patients with macular degeneration. This provides interesting insights into differing levels of digital engagement and the availability of relevant local online communities in each country. When digitally listening to patients’ voices, active platforms or online communities should be included to collect meaningful data pools.

Thirdly, the high level of digital engagement by patients with AMD aged under 50 years, provides a valuable opportunity to understand the specific needs of younger patients better. These younger patients are more likely to be working, have dependents, and may face long-term quality of life impacts of their condition. We found that younger patients are more likely to actively seek anti-VEGF treatment; however, they also mentioned the challenge of access to National Health Insurance to cover the cost of treatment. This could be an opportunity to understand the needs of patients under 50 years, who are not eligible for insurance [24, 25]. Understanding the concerns voiced by these patients may allow healthcare systems to respond earlier to their unmet needs.

Lastly, this study comprehensively collected unsolicited patients’ voices directly across the patient journey, which included criteria to select treatment centres or doctors, treatment expectations and concerns, treatment burdens such as tolerability and financial burden, and treatment persistence. This provided an overall picture of the needs of the patients and how key patient-centric solutions could be created by all involved parties of the healthcare system.

## Conclusions

Semantic-NLP was used to identify the real-world voices of patients with AMD in Korea and Taiwan, with findings similar to those from qualitative interview studies. This demonstrates that Digital Listening using Semantic-NLP can provide real-world insights from large amounts of internet data quickly and with low human labour cost. It can provide healthcare companies with the information required to respond to patients' unmet needs for effective and safe treatments and develop solutions to allow a better quality of life for patients throughout the product lifecycle.

## Supplementary Information

Supplementary Material 1.

## Data Availability

The datasets used and/or analysed during the current study are available from the corresponding author on reasonable request.

## Abbreviations

AI: Artificial intelligence
VEGF: Vascular endothelial growth factor
AMD: Age-related macular degeneration
NLP: Natural language processing
OBIE: Ontology-based information extraction
SML: Social media listening
