# Supplementary material for: Real-world insights of patient voices with age-related macular degeneration in the Republic of Korea and Taiwan: an AI-based Digital Listening study by Semantic-Natural Language Processing
Source: BMC Med Inform Decis Mak. 2025 Mar 18;25:137. doi: 10.1186/s12911-025-02929-5 (PMC11916980; doi:10.1186/s12911-025-02929-5)
Supplement: Supplementary file 1 — Supplementary Material 1. [file 12911_2025_2929_MOESM1_ESM.docx]

**SUPPLEMENTARY TABLE: Full list of AMD treatments included in this study**

| **Anti-VEGF therapy** | Faricimab-svoa (Vabysmo)– Roche |
| --- | --- |
|  | Ranibizumab (Susvimo) – Roche |
|  | Brolucizumab (Beovu) – Novartis |
|  | Pegaptanib (Macugen) – Eyetech / Pfizer |
|  | Ranibizumab (Lucentis) – Novartis |
|  | Aflibercept (Eylea, Zaltrap) – Bayer |
|  | Conbercept (Lumitin) — Chengdu Kanghong Biotech, China |
|  | KSI-301 — Kodiak Sciences |
|  | Bevacizumab (Avastin) — Roche |
|  | IONIS-FB-LRx, RG6299 – Ionis Pharmaceuticals |
|  | Triamcinolone |
|  | Dexamethasone - Ozurdex |
| **Biosimilars** | Ranibizumab biosimilar (Amelivu) - Samsung Bioepis Co., Ltd. |
|  | Ranibizumab biosimilar (Cimerli) - Coherus BioSciences |
|  | Ranibizumab biosimilar (Byooviz) - Biogen |
|  | Ranibizumab biosimilar (Ranivisio/Ongavia) – Teva |
|  | Ranibizumab biosimilar (Razumab) - Intas Pharmaceuticals |
|  | Ranibizumab biosimilar (Ranizurel) - Reliance Life Sciences Ltd. |
|  | Ranibizumab biosimilar (Ranibizumab BS 1) - Senju Pharmaceutical |
|  | CKD-701, ranibizumab biosimilar - Chong Kun Dang Pharmaceutical |
|  | GNR-067, ranibizumab biosimilar - AO GENERIUM |
|  | LUBT010, ranibizumab biosimilar - Lupin Ltd. |
|  | Ranibizumab biosimilar (Xlucane) - Xbrane Biopharma |
|  | SCD411, aflibercept biosimilar - Sam Chun Dang Pharm. Co. Ltd. |
|  | SOK583A1, aflibercept biosimilar - Sandoz, Novartis |
|  | SB15, aflibercept biosimilar - Samsung Bioepis Co., Ltd. |
|  | FYB203, aflibercept biosimilar - Bioeq GmbH |
|  | ABP 938, aflibercept biosimilar - Amgen, Parexel |
|  | 9MW0813, aflibercept biosimilar - Mabwell (Shanghai) Bioscience |
|  | AVT06, aflibercept biosimilar - Alvotech Swiss AG |
|  | MYL1710, aflibercept biosimilar - Momenta Pharmaceuticals and Mylan |
|  | TAB014, bevacizumab biosimilar - Zhaoke (Guangzhou) Ophthalmology Pharmaceutical Ltd. |
|  | Bevacizumab biosimilar (Lumiere) - Laboratorio Elea Phoenix S.A. |
|  | ONS-510, bevacizumab biosimilar - Outlook Therapeutics |
|  | ALT-L9, aflibercept biosimilar - Alteogen Inc. |
| **Other treatments** | photodynamic therapy (hereinafter - PDT) |
